# Supplementary material for: Are carbon emissions trading and green financial instruments synergistic? -Comprehensive quantitative research based on content analysis
Source: PLoS One. 2024 Mar 7;19(3):e0298601. doi: 10.1371/journal.pone.0298601 (PMC10919601; doi:10.1371/journal.pone.0298601)
Supplement: S1 Data — (ZIP) [file pone.0298601.s001.zip › Data/地级市Z_F工作报告.zip!/╜¡╦╒/─╧═¿╩╨/└┤╫╘╣1⁄2╓┌║┼í╛┬φ┐╦╩2╛▌═°í┐_2014.docx]

各位代表：

　　现在，我代表市人民政府向大会作工作报告，请予审议，并请市政协委员和其他列席人员提出意见。

　　一、2013年工作回顾

　　过去的一年，我们认真学习贯彻党的十八大和十八届二中、三中全会精神，在省委、省政府和市委的正确领导下，紧扣主题主线，坚持稳中求进，奋力落实“八项工程”、实现“八个领先”，统筹推进稳增长、调结构、促改革、惠民生，较好完成了全年目标任务。预计地区生产总值5039亿元，增长11.8%。财政总收入1217亿元，地方公共财政预算收入486亿元、增长15.8%。预计，固定资产投资3299亿元，增长20.8%；社会消费品零售总额1927亿元，增长12.8%；外贸进出口总额298亿美元，增长13.4%；注册外资实际到账25亿美元；城镇居民人均可支配收入31059元，农村居民人均纯收入14754元，分别增长9.8%和11.5%；各项存款余额7542亿元，居民储蓄存款余额4151亿元、增长15.1%；居民消费价格涨幅2.2%；城镇登记失业率2.15%；全社会研发投入占GDP比重2.35%；完成省下达节能减排任务。

　　（一）经济发展保持稳中有进。采取有效措施促发展，增长质量和效益稳步提升。政策支撑力度加大。完善保持经济平稳较快发展的“七个方面”措施，制定优化产业布局、推动开发园区转型和科技“三创”载体建设、促进商务稳中求进以及信息基础设施建设、做大做强企业三年行动计划等30多项政策意见。支持实体经济发展，出台市区重大项目落户零收费政策，全市停征、减收46项涉企收费，推进“营改增”试点，为企业减负27.7亿元；新增贷款666亿元，中期票据、短期融资券等债券融资98亿元，中小企业集合票据“区域集优”实现零的突破。产业转型升级加快。六大主导产业产值突破1万亿元，家纺、电动工具分别建成国家级、省级新型工业化示范基地。新兴产业产值3404亿元，海工、光伏等10条特色产业链初具规模，空港、光电等特色产业园加快建设。服务业增加值2093亿元、占GDP比重41.5%，市级以上服务业集聚区达50家，新增海安商贸物流园、开沙岛旅游度假区等一批省级园区和基地，首家世界500强区域总部落户。成为国家智慧城市和中欧绿色智慧城市试点，新增国家电子商务集成创新试点项目2个、省级“两化”融合示范试点企业65家。建筑业施工产值4350亿元。主营业务收入超百亿元工业、商贸、建筑企业（集团）达14家。创新驱动能力增强。国家创新型试点城市建设和全国质量强市示范城市创建扎实推进，高新技术产业产值占规模以上工业比重42.4%。新增2家国家级企业技术中心、3家省级院士工作站、49家省级企业技术（研究）中心。成立南通产业技术研究院。国家级南通高新区获批。全市新增“三创”载体面积57万平方米。引进国家“千人计划”人才26名、省级人才资助对象68名。成为省级科技金融示范区，创投、股权投资机构新增27家、总数达72家，新兴产业创投引导基金正式运作，高端装备创投和科技创业风投基金成立。

　　（二）江海联动成效加速显现。以高水平规划引领陆海统筹、江海联动，制定沿海开发三年行动计划，沿海地区发展实现新突破。重大项目顺利实施。恒力新材料、招商局海工等百亿级产业项目部分竣工投产。洋口港江苏沿海首个10万吨级石化码头主体工程基本建成，通州湾建港起步工程开工，海门港、如皋港集装箱航线开通，吕四国家级中心渔港新港区建成启用，南通港吞吐量突破2亿吨。沪通铁路和通苏嘉城际铁路长江大桥工程进入实质性开工阶段，宁启铁路复线电气化改造线下工程完成，临海高等级公路全线贯通，通洋高速、通州湾快速通道等项目加快实施，连申线航道南通段通航。全市围垦滩涂5.3万亩。载体功能不断突破。南通开发区平台建设成效明显，综合保税区正式封关运作。如皋、海门开发区进入“国家队”，国家级开发开放平台达6个。省级开发区和滨海特色园区产城融合步伐加快，南通滨海园区开发建设快速推进。兴东机场开放列入国家审理计划，电子口岸上线运行。融合发展加快推进。跨江合作园区达12家，苏通、锡通科技产业园和上海市北高新（南通）科技城功能提升，中奥苏通生态产业园启动建设。对接上海自贸区，组织现代物流、加工贸易与后台服务专题招商。

　　（三）改革开放活力增强。省委、省政府印发南通陆海统筹发展综合配套改革试验区总体方案。重点领域改革不断加强。国有资产保值增值率达105%。投融资体制改革扎实有效，引进汇丰、恒丰、光大银行，组建2家融资租赁公司，新增场外市场挂牌企业12家。资源要素配置改革积极实施，成为全国海域使用管理创新试点市，城镇低效用地再开发试点工作启动。市区城建管养、市容管理等体制调整到位，“区镇合一”管理体制改革深化，对市级中心镇下放经济管理权限。教育、医疗、文化和事业单位等社会领域改革深入推进。开放型经济量质并举。引进超亿美元项目22个，服务业利用外资占比达30%。获批国家内外贸结合试点，市级以上出口基地出口占比达60%。服务外包执行额增长35%。外经主要指标保持全省第二。成功举办中国航海日大会、南通江海国际博览会、台商产业转型升级峰会。外事、侨务、港澳和对台事务不断加强。民营经济转型势头良好。民营经济增加值、入库税金占比分别提高1个、2.5个百分点，引进市外民资570亿元。新培育百亿级特色产业板块2个。新增著名商标33件、省级名牌产品89个。中国中小企业总部基地落户。

　　（四）城乡统筹力度加大。统筹五级城乡空间体系建设，城乡发展一体化加速推进。中心城市实现新提升。开展60项专项规划编制。158个城建项目完成投资218亿元。老通启路、东方大道快速化改造等项目竣工，江海大道东延主线通车，市区快速路总里程达80公里，“102030”交通畅通目标基本实现。完成32个骨干道路和23个支路项目。汽车客运东站建成试运营。新城区一批商务、金融、文化等功能性项目竣工，观音山新城起步区开发基本完成。南部新城、市北新城产城融合度进一步提高，通州区与主城区融合发展加快。西寺、唐闸等历史街区保护修缮稳步推进，完成“城中村”、老小区改造整治等项目41个，获批国开行全国第一单棚户区改造专项贷款100亿元。市区新建2个综合性公园、6个小游园和一批道路绿化带。城镇化建设迈出新步伐。全市城镇化率59.9%。5个县（市）城中等城市框架进一步拉开，海安完成城市总体规划修编。19个市级中心镇总体规划编制完成，67个重点产业和244个民生项目顺利推进。一批特色镇加快培育。农业现代化工程取得新成效。粮食生产实现“十连增”。新增设施农（渔）业13.2万亩。“三资”农业项目投资230亿元。家庭农场、“全托管”等新型农业经营主体快速发展，农民合作社入社农户194万户。水利建设完成投入34亿元，引江区域供水和农村安全饮水工程成果得到巩固。新建高标准农田22万亩。

　　（五）社会建设持续加强。落实民生幸福工程，促进城乡居民持续增收，完善社会建设“十大体系”，地方公共财政预算支出中民生投入比重56.1%，10件40项为民办实事项目全面完成（见附件一），28项重点民生工作推进成效明显。就业和保障水平不断提高。新增就业9.2万人，帮扶创业1.2万人。五大社会保险覆盖率稳定在97%以上，企业退休人员养老金、新农合筹资、城乡居民基础养老金和最低生活保障平均标准均有较大幅度提高，城镇居民大病保险不设报销封顶线。社会救助、扶贫攻坚扎实开展。基本公共服务体系加快构建。成为首批全国义务教育发展基本均衡地级市，省级优质幼儿园占比达75%，紫琅学院升本通过专家组评估，南通高师升专获批。环濠河博物馆群成为全国公共文化服务示范项目，唐闸近代工业遗存“申遗”全面启动，城市文明程度指数测评居全国全省前列。镇村医疗卫生机构一体化管理有力推进，实现基本药物制度全覆盖。通大附中、市妇幼保健院、图书馆新馆等公共服务项目投入使用或基本建成。市区安置动迁户1.9万户，全市竣工各类保障性住房11886套（间）。每千名老人拥有养老床位27张，居家养老服务网络初步构建。完成市区菜市场标准化改造三年行动任务。市十运会暨首届全民健身运动会成功举办。人口计生服务进一步加强。社会大局保持和谐稳定。平安法治南通建设不断深化，“一综多专”大调解体系、社会服务管理综合平台等更趋健全，公众安全感、刑事案件破案率位居全省前列。村（居）委会换届选举完成，社区服务功能增强，省级城市、农村和谐社区建设达标率分别达80%、78%。食品药品监管和信访、民族、宗教等工作得到加强。安全生产形势总体平稳。国防动员、征兵工作成效明显，军政军民关系更加融洽。

　　（六）生态文明建设扎实推进。制定生态红线区域保护规划和生态文明建设三年行动计划，获评全国十佳生态文明建设示范城市。海门、如皋、如东、通州通过国家生态县（市）区创建验收，全市通过技术评估。资源节约成效明显。完成120个清洁生产项目，新增1家国家级循环经济标准化试点、2家省级循环化改造示范试点园区。连续22年保持耕地占补平衡。成为国家级节水型社会建设示范市。铁腕治污深入开展。实施240个减排工程，重点行业标准化整治、化工园区生态化创建加快推进。秸秆禁烧和综合利用取得新成效，燃煤锅炉和黄标车淘汰、油气回收改造等控煤降尘措施进一步落实，PM2.5监测实现县（市）区全覆盖。市区水资源综合管理机制形成，城乡河道疏浚整治年度任务完成，应急水源项目一期建成，饮用水源地保护加强。城乡面貌持续改善。完成城市环境综合整治年度任务，村庄环境整治深入推进。市区新增公园绿地427公顷，全市新增绿化造林14.9万亩。

　　各位代表，一年来，我们积极转变政府职能，服务型政府建设进一步强化。政府效能得到提升。推进简政放权，深化行政审批“三集中、三到位”，审批事项削减25%，工商企业注册登记并联审批等制度启动实施，网上审批全面推开。市政务中心、公共资源交易中心、综合电子监察中心和“12345”政府服务热线有序运行。政府工作推进、协调、督查三大体系作用积极发挥，市政府100项年度重点工作全面完成。依法行政逐步深化。行政权力网上公开透明运行深入实施，政府信息公开范围持续拓展。综合执法机制加快构建。自觉接受市人大及其常委会的法律监督、工作监督和市政协的民主监督，认真办好市人大关于推进中心镇建设促进城乡统筹发展的代表议案和市政协关于完善社会保险体系推进民生幸福工程、推动生态环境建设加快打造美丽南通的建议案，全面完成人大代表建议、政协提案办理任务。廉政建设切实加强。严格执行中央改进工作作风、密切联系群众八项规定和省、市委十项规定，扎实开展“三解三促”，认真组织“三项清理”。厉行勤俭节约、反对铺张浪费，市级机关公用经费削减10%。切实加强效能监察、执法监察、廉政监察和审计监督，内部审计制度更加完善。

　　各位代表，过去一年，各项成绩的取得，是省委、省政府和市委正确领导，市人大、市政协监督支持，全市人民团结一致、攻坚克难、奋勇拼搏的结果。我代表市人民政府，向全市人民，向人大代表、政协委员和离退休老同志，向市各民主党派、工商联、各人民团体和社会各界人士，向驻通人民解放军、武警官兵和公安干警以及部省驻通单位，向关心和支持南通发展的海内外朋友，致以崇高的敬意和衷心的感谢！

　　各位代表，我们也清醒认识到发展中的差距和工作中的不足。产业结构性矛盾仍较突出，新兴产业和服务业规模还不大，自主创新能力还不强，领军企业、重特大项目、高端科研院所和人才还不多。深化改革的任务十分繁重，资源要素市场化配置水平还不高。城镇化质量和水平有待提升，城乡二元结构仍较明显。环境治理和生态保护任务艰巨，雾霾污染防治缺乏有效手段。民生工作还存在一些薄弱环节，城乡居民收入增长趋缓，部分群众生活仍较困难，公共服务体系和能力与群众多元化需求仍有差距。社会不稳定、不和谐因素仍然较多。政府自身建设还不适应新形势的要求，职能转变还不到位，政务服务效能和群众工作能力还有较大提升空间，干部作风、反腐倡廉建设方面的问题还不同程度存在。对此，我们将采取有效措施，认真加以解决。

　　二、2014年工作要求

　　今年是全面深化改革的第一年，是全面完成“十二五”规划目标任务的关键一年。世界经济进入深度调整期，既有缓慢复苏态势，又有诸多不确定因素；我国既有总体向好的宏观环境，又有日益增多的“两难”问题。我市人均GDP跃上1万美元新台阶，处于扩量提质、创新转型同步推进阶段，既要更加敏锐抢抓江苏沿海开发、长三角一体化发展和上海自贸区、“两带一路”建设等战略叠加机遇，用好陆海统筹发展综合配套改革和沪通铁路建设等独特发展机遇；又要更加有效应对区域竞争压力和经济下行风险，破解中等收入阶段发展难题。我们必须始终保持清醒头脑，坚持问题导向和底线思维，进一步增强忧患意识和责任意识，稳中有为，提质增效，开创“两个率先”新局面。

　　今年政府工作的总体要求是：深入贯彻落实党的十八大和十八届二中、三中全会以及中央经济工作会议精神，按照省委十二届六次全会、省经济工作会议和市委十一届六次全会部署，围绕争当苏中新一轮发展“领头雁”、建设长三角北翼经济中心的目标，坚持稳中求进工作总基调，把改革创新贯穿于经济社会发展各个领域各个环节，以“八项工程”为主抓手，以转型升级为主攻方向，以科技创新为核心驱动力，加快转方式调结构，着力创建陆海统筹发展综合配套改革试验区，着力激发市场活力，着力改善民生，切实提高经济增长的质量和效益，加强基本公共服务体系建设，促进经济持续健康发展、社会和谐稳定。

　　今年经济社会发展的预期目标是：地区生产总值增长10%；地方公共财政预算收入增长12%；固定资产投资增长18%；社会消费品零售总额增长13%；外贸进出口总额增长5%；城镇居民人均可支配收入、农村居民人均纯收入均增长11%左右；居民消费价格涨幅控制在3.5%左右；城镇登记失业率控制在4%以内；全社会研发投入占GDP比重达到2.4%；万元地区生产总值能耗下降、主要污染物减排完成省下达任务。

　　实现上述目标，需要我们牢牢把握：

　　（一）在加快转型升级中保持经济持续健康发展。坚定不移以转促进、以进促稳，抓增量把握质态，调存量把握结构，壮大新产业、引进新技术、集聚新人才，促进有效需求充分释放、创新能力持续增强、总量质量同步提升。

　　（二）在加快陆海统筹江海联动中放大融合发展特色和优势。强化陆域与海洋统筹开发，加快优江拓海、陆海互动、特色发展，把沿江开发优势放大到沿海、江海联动优势放大到长江流域，促进融入苏南、融入长三角核心区第一方阵。

　　（三）在加快改革开放中提高经济国际化水平。全面深化改革，以改革推动开放、以开放倒逼改革，构建系统完备、科学规范、运行有效的体制机制，促进民资、外资、国资等多种所有制经济竞相发展，企业、载体、城市、人才等国际化加速推进。

　　（四）在加快新型城镇化中推动城乡发展一体化。以科学规划为引领、人的城镇化为核心、体制机制创新为关键，推动大中小城市、特色镇和规划布点村庄（新型社区）协调发展，促进形成以工促农、以城带乡、工农互惠、城乡一体的发展格局。

　　（五）在加快生态文明建设中增强可持续发展能力。坚持经济效益、社会效益与生态效益并重，更大力度推进资源保护、环境治理和生态建设，促进经济发展与生态宜居协调融合、都市风貌与田园风光交相辉映、人与自然和谐共生。

　　（六）在加快社会建设中增进民生幸福和谐。立足当前办实事、着眼长远建体系，既积极解决老百姓最关心最直接最现实的利益问题，又注重完善保障改善民生和创新社会治理的制度安排，促进发展成果更多更公平惠及全体人民。

　　三、2014年主要任务

　　做好今年工作，我们将坚持以改革创新的精神推进全面、协调、可持续发展，对更有质量、更高效益、更加公平的增长毫不放松，稳中求进、进中求好、又好又快。着重抓好十个方面：

　　（一）以构建现代产业体系为方向，更大力度优化结构。围绕调高调强调轻调优，推动产业高端化、智能化、服务化发展。

　　强化先进制造业支撑。大力推进新型工业化，加快培育一批市场竞争力强、技术密集度高的产业集群。做大做强主导产业。突出抓好船舶、家纺等产业的布局优化、转型升级，提高六大“千亿级”板块的集聚度和附加值；推动建筑业向模块化、数字化、低碳化发展。着力发展新兴产业。围绕“3+4”重点领域，深入实施“310”工程，创建国家战略性新兴产业国际合作区、海洋工程国家创新高地和光伏规模化应用示范区，新兴产业产值超过3700亿元。加快构建特色产业链。以特色园区、基地建设为抓手，大力布局发展全产业链，促进过剩产能消化和价值链、创新链、供需链整合提升。全力实施大企业战略。鼓励企业改造技术、制定标准、创新商业模式，打造一批有市场话语权的领军企业。

　　加快现代服务业发展。把集聚区建设和平台经济培育作为重点，提升服务业发展能力与水平。突出“7+3”重点领域，实施168个服务业重点项目，服务业增加值占GDP比重达到42%。制定新兴服务业发展规划，在总部经济、工业设计、数据管理、供应链管理、知识流程外包等方面求突破。做大做强现代物流、金融、科技、商务服务等生产性服务业。培育信息消费和养老、健康等服务业，提升商贸流通服务水平，增强消费的基础作用。丰富旅游产品和业态，加快把旅游业建成支柱产业。

　　提高农业现代化水平。稳定粮食生产，抓好“菜篮子”工程。加快农业结构调整，壮大优质粮油、蔬菜园艺、特种养殖、现代渔业等主导产业，培育花卉苗木、生态休闲等特色产业，发展设施农（渔）业9万亩。鼓励农业适度规模经营，加快项目农业建设，有序推进农村土地综合整治和承包经营权流转。引导工商资本合理投资农业，新增农（渔）业规模园区15个、市级以上龙头企业40家。完善新型农业经营体系，培育“全托管”等新型经营主体，新增示范性家庭农场20家、农民合作社100家。加强农业科技创新，提升种苗研发、农业机械化和农技推广水平。

　　发挥信息化引领作用。推进国家智慧城市和中欧绿色智慧城市试点，实施“宽带、光网、无线南通”和“三网融合”工程，完成信息基础设施投入25亿元。促进与智慧城市相关的信息产业、信息服务业加速发展，争创省级“两化”融合示范区，在电子商务、云计算、大数据、物联网等领域引进一批龙头企业，培育一批示范园区。提升农业农村和公共服务信息化水平，建设智慧家庭、智慧社区、智慧交通、智慧民生等应用示范项目。

　　（二）以增强自主创新能力为核心，建设创新型城市。着力完善开放式、网络化、集聚型、高效率的区域创新体系。

　　促进创新要素向企业加速集聚。围绕培育“五自”创新型企业，深入实施高新技术产业“双倍增”工程，高新技术产业产值超过5500亿元。健全企业主导产业技术研发创新的体制机制，支持家纺、海工、建筑等领域优势企业抢占核心技术和标准高地，新增省级以上研发机构30家。鼓励企业联合开展技术攻关，承接一批国家科技重大专项和省重大科技成果转化项目。落实好重大装备首台套应用等政策，引导企业加大研发投入。

　　促进产业与科技人才紧密结合。健全产学研协同创新机制，实施300个联合攻关项目，促进技术链、产业链双向融合。强化应用研发导向，鼓励重点地区、龙头企业瞄准产业技术前沿，与科研院所、高等院校共建创新平台。加强新技术示范推广，在省级以上开发区规划建设一批产业化基地。以人才高地引领产业高地建设，深入实施产业人才发展“312”行动计划，健全人才招引、培养和服务工作体系，新引进国家“千人计划”人才20名。

　　促进创新载体和环境持续优化。支持南通高新区加快科技城等特色平台建设，推动省级高新区等创新园区形成“一区一主导产业”。加快南通产业技术研究院建设，尽快突破1-2个产业的关键技术和成果产业化。新建“三创”载体面积45万平方米。完善共性技术研发、成果中试等平台，提升科技公共服务水平。推进知识产权示范市和质量强市示范城市建设，建立健全知识、专利、技术、品牌、管理等要素的创新激励机制。构建多层次科技投融资体系，大力发展科技金融，新设立创投和股权投资机构15家。

　　（三）以实施陆海统筹发展综合配套改革为重点，增创体制机制优势。坚定不移解放思想、敢于担当、先行先试，把陆海统筹发展综合配套改革作为全面深化改革的重点，分门别类、扎实推进，形成特色、创造品牌，全力突破、走出路子。有序、协调、稳步推进其他各项改革，加快体制机制创新。

　　深化经济体制改革。发挥市场配置资源的决定性作用，激发各类市场主体活力。推进国有企业改革。促进国有企业股权多元化，优化国有资本投向，健全国有投融资平台。推进民营经济改革。降低门槛，放宽准入，更大力度发展民营经济，鼓励“个转企、小上规”，民营经济增加值占比达到64%。以技术、品牌、管理创新推动民营经济转型升级，深入实施“三名”工程，新增100家民营高新技术企业，创建1个全国知名品牌示范区。推进金融改革。积极引进银行、证券、基金、信托等金融机构，发展地方金融、民营金融、小微金融和“商圈贷”、“供应链融资”等新型融资方式。构建多层次资本市场，推动更多企业上市和在“新三板”挂牌，直接融资规模达到100亿元。推进统筹陆海资源改革。深化海域使用权市场化改革，建设滩涂综合开发试验区，建立健全城乡统一的建设用地市场。推进财税体制改革。完善财政专项资金配置和绩效管理制度，深入推进“营改增”试点。加强地方政府性债务规范管理和风险防范。

　　深化行政体制改革。以简政放权为核心，创新行政管理方式，正确处理政府与市场关系。落实改革举措，稳步推进机构改革，加快事业单位分类改革，制定政府购买服务指导意见。拓展行政审批“三集中、三到位”内涵，加大审批事项“减、转、放、免”力度，确保行政权力放得下、接得住、运转得好；加强并联审批和网上审批，探索审批服务市场化改革，不断提高审批效率。营造开放有序的市场环境，推进工商注册制度便利化，在综合保税区等特殊区域先行先试，积极探索负面清单、“先照后证”投资管理模式。下放管理权限，激活县（市）区和重点区域发展潜能。

　　深化城乡体制改革。围绕“八个一体化”，强化政策设计，逐步破除城乡二元结构。加快户籍制度改革，推行居住证制度，促进农业转移人口有序实现市民化。稳步推进“三集中、三置换”，制定基本公共服务均等化规划，鼓励有条件的地区实施城乡社会保障并轨，促进城乡要素平等交换和公共资源均衡配置。坚持和完善农村基本经营制度，推进农村土地、产权等制度改革，赋予农民更多财产权利。加强农村金融服务，化解农村信贷抵押难题，支持农村金融机构发展，鼓励合作社开展信用合作。

　　深化社会体制改革。整合资源，创新手段，使社会既充满活力又和谐有序。加快教育领域综合改革。抓好高职教育、中考改革国家试点项目，探索建立管办评工作新机制。统筹医药卫生体制改革。深化县级公立医院和基层医疗卫生机构综合改革，建立社区卫生服务机构与综合性医院双向转诊制度。完善文化管理体制。深化国有经营性文化单位改革，培育多层次文化市场主体。创新社会治理方式。健全“稳评”、“安评”、“环评”和诉求表达、矛盾调处、群众自治等机制，构建多层次、宽领域、规范化的大调解组织网络体系，推行“阳光信访”和信访法治化。

　　（四）以实现沿海开发全省领先为目标，纵深推进江海联动。加快沿海科学发展、沿江转型发展、江海联动发展，努力成为苏中融合发展先行区、接受上海和长三角核心区辐射的先导区。

　　强化优江拓海。完善沿江沿海规划体系，促进沿江“腾笼换凤”和沿海“筑巢引凤”有机结合。落实省沿江地区转型发展五年推进计划，推动市区等重点区域整合、集聚、提升，加快产业梯度布局和转移。用好省进一步促进沿海地区科学发展的若干政策，对接省“六大行动”计划，提高沿海港产城融合发展水平。制定海洋经济发展意见，引导石化、能源、重装备、现代物流等临港产业发展，培育海洋战略性新兴产业和涉海服务业，提升现代海洋渔业，海洋经济生产总值达到1550亿元。推动沿江沿海特色园区错位发展，打造一批应税销售超百亿元的产业园区。

　　强化项目带动。发挥投资关键作用，抓好总投资4700多亿元的160个市级重大项目，力争完成投资720亿元。建设洋口港区、吕四港区、通州湾航道和码头。建设沪通铁路和通苏嘉城际铁路长江大桥工程以及海启高速，建成宁启铁路复线电气化改造、通洋高速一期等工程，推进锡通高速、宁启铁路二期、崇海大桥等前期工作。统筹电力、信息等基础设施向沿海布局。

　　强化区域合作。加大与上海、苏南共建园区力度，促进苏通、锡通科技产业园等跨江合作园区特色发展，支持上海市北高新（南通）科技城创建省级高新区，导入更多优质项目、资源要素和发展政策。主动参与“两带一路”建设，大力推进江海河、空铁水联运项目，探索与长江中上游、中西部地区重点城市建立有效合作机制，促进产业协作、要素流动与共同发展。

　　（五）以对接上海自贸区和加快走出去为突破口，构建开放型经济新格局。抢抓新一轮对外开放机遇，促进“六外”并举，增强国际经济合作竞争新优势。

　　推动引进来与走出去更好结合。着力促进国内外要素有序自由流动、市场深度融合。主动学习、接轨、配套、服务上海自贸区。充分发挥综合保税区独特功能，积极承接现代物流、加工贸易、后台服务、分拨分销等“溢出项目”，发展离岸金融、跨境结算、融资租赁，强化载体、功能、平台对接，复制推广体制机制创新经验。更大力度引进外资。强化区域性、龙头型、产业链项目招商，鼓励外资企业增资、扩股、重组，提高超亿美元项目和新兴产业、服务业项目比重，注册外资实际到账25亿美元。落实外贸稳增长促转型政策。扩大重点行业、企业和基地出口，促进内外贸一体化发展，增加关键设备、资源性产品进口，提升以技术、品牌、质量、服务为核心的外贸竞争力。推动走出去转型升级。加快对外经济由劳务输出向工程总包、国际贸易、项目投资、园区开发转变，打造示范性境外产业集聚区和经贸合作区，利用国际市场消化过剩产能。

　　推动开发园区创新转型发展。实施开发园区转型升级工程，促进各开发区在全省开发区科学发展综合评价中争先晋位。加快国家级开发园区功能提升。推动南通开发区以“5+3”特色园区为依托，引进高端项目，发挥示范引领作用。推进4个新批国家级开发园区形态和功能开发，增强集聚、服务和创新发展能力。加快省级开发园区升级扩容。引导省级开发园区壮大主导产业，创建一批创新型开发园区。推动启东、如东等省级开发区升级，支持有条件的园区创建省级开发园区。加快特色开发园区建设。做强一批特色“区中园”，打造承接服务外包、集聚特色产业、服务创新创业的专业化园区。加快发展海关特殊监管区。

　　推动开放平台建设加快突破。在投资、贸易便利化等平台建设上找准切入点，提升开放服务功能。做强通关服务和物流平台，争取“三个一”联合通关试点，完成电子口岸二期开发，推动机场、海港获批国家一类开放口岸，加快保税物流中心、保税仓库申报。推进贸易方式创新平台建设，争取国家市场采购贸易方式试点，力争获批跨境贸易电子商务试点。健全大宗商品展销平台，加快LNG、油品和棉花、食用油、海产品等交易平台建设。

　　（六）以提高首位度为着力点，增强中心城市辐射带动力。开展新一轮城市总体规划修编研究，引领城市经济发展、功能优化、品质提升、为民服务，发挥中心城市龙头作用。

　　推进组团开发。以快速路网为引领，强化城市组团化、差异化、特色化发展。加快通沪大道两侧开发，提升观音山新城核心区形象，促进新城区繁荣，力争22个项目竣工或主体封顶。实施一批功能性项目，提高南部新城国际化发展水平。以北大街和江海大道为轴线，加强市北新城成片、混合开发。加快与通州区连接通道建设，促进东部新城和南通滨海园区发展提速。统筹重要功能片区建设，推动“五水”地区开发，打造“两河两岸”景观带，促进五山景区整合提升。抢抓国家支持棚户区改造机遇，加快“城中村”、老小区改造整治，稳步实施寺街、唐闸、陆洪闸、天生港等历史文化街区、镇区保护改造。

　　壮大城市经济。以城市集聚经济扩大规模为方向，优化要素投入和产业政策引导，加快“优二进三”，大力发展2.5产业、平台经济、交易中心等生产性服务业，引进和培育总部基地、研发中心、楼宇经济、金融后台、文化创意等高端业态。加强商业金融文化集聚区、科技创业社区、城市综合体等现代产业载体规划建设，积极发展特色鲜明的区域性商圈、商贸中心和商业街区。

　　完善基础设施。以基础设施项目为重点，实施总投资超300亿元的242个城建项目。加快完善道路网络，推进长江路西延等快速路项目和重要节点互通工程建设，实施园林路北延等63个骨干路和一批支路项目。促进公共交通发展，新增公交车150辆，公交运营线路突破100条，做好城市轨道交通前期工作，建成慢行道路80公里。健全城市水电气和信息等服务体系，提高引江区域供水能力，加强地下管网建设和管理。

　　提升城市品质。以“九整治、五规范、一提升”为重点，深入开展城市环境综合整治。进一步完善城市管理长效机制，加快形成以区为主、条块联动的管理格局。推进国家生态园林城市创建，优化濠河周边环境，打造通沪大道、东快速路等28条道路绿化带，因地制宜建设一批公园绿地和小游园，合理布局文化休闲、体育健身等设施，增加城市公共绿色空间。

　　（七）以推动城镇化特色发展为主导，促进城乡共同繁荣。积极、稳妥、扎实推进新型城镇化和城乡发展一体化，加快构建具有南通特色的五级城乡空间体系，提高城乡统筹发展水平。

　　优化城镇化布局。完善市域“一主三副多点”的城镇结构，促进县（市）城、中心镇、特色镇加快发展，力争全市城镇化率提高2个百分点。推动县（市）城进一步增强功能，集聚人气。按照全面城市化型、产业推进型、交通枢纽型、特色发展型等分类，因地制宜、扎实推进19个市级中心镇和8个沿海前沿“区镇合一”的城镇建设，增强对区域发展的带动力。鼓励小城镇个性化发展，建设一批工业商贸强镇、文化旅游名镇和绿色智慧城镇。

　　提升城镇化质量。坚持以人为本、产城融合，加强基础设施建设、产业培育和公共服务，促进城镇化健康发展。完善县（市）域路网，推进一批干线公路项目，积极发展镇村公交。加强建制镇污水处理设施建设、管网配套和运行管理。支持各地抓好具有区域特色的产业项目和基地，提高重点城镇产业集聚度。加大资源配置力度，加快教育、文化、卫生等公共资源在城镇合理布局。稳步推进社保等基本公共服务城镇常住人口全覆盖。

　　夯实城镇化基础。加大强农惠农富农政策力度，更好履行“三农”工作职责。强化规划布点村庄建设和农村新型社区功能配套，引导农民自愿集中居住。加强以水利为重点的基础设施建设，新建高标准农田20万亩，推进农村公路提档和江海堤防提标工程，开工建设九圩港复线船闸和提水泵站。深化农村环境“四位一体”长效管理，村庄环境整治通过省级验收。实施“美丽乡村”行动和生态循环农业发展试点，展现农村独特风貌。

　　（八）以强化保护治理并重为抓手，打造生态宜居家园。实施生态文明建设三年行动计划，创造更好的生产生活环境。

　　推动绿色增长。多管齐下挖掘节能减排潜力，深化重点行业标准化整治和各类园区生态化创建，加大污水处理、固废处置等基础设施投入。大力发展生态、循环、低碳经济，加快淘汰落后产能，实施化工等产业环保负面清单管理。完善资源节约集约使用制度，加强土地、海域用途管制和保护开发。

　　强化环境治理。狠抓以集中式水源地为重点的水污染防治，健全河长制、段长制，加强城乡河道、重点水系治理，持续优化水环境。狠抓以PM2.5为重点的大气污染防治，落实秸秆禁烧与综合利用、燃煤锅炉改造、机动车尾气和扬尘管控、工业废气治理等措施，减少雾霾污染。狠抓以耕地等为重点的土壤污染防治，强化工业点源、农业面源和生活污染源整治，改善土壤质量。

　　加强生态保护。推进“绿色南通”建设，构建“两沿三园”绿化空间体系，新增造林12万亩。严格落实生态红线区域保护规划，优化主体功能区布局和国土空间开发形态，加强江海河水网、自然湿地、生物多样性保护。建立陆海统筹、江海联动的生态系统保护修复机制，争创国家海洋生态文明示范区。

　　落实制度保障。实行最严格的生态环境源头保护、总量控制、损害赔偿、责任追究等制度，制定碳排放控制计划。建立资源有偿使用和生态补偿制度，推进排污权交易等试点工作。健全污染物排放监管制度，强化污染防治区域联动。推进国家生态市等绿色创建，完善环保社会监督、科普宣传等机制。

　　（九）以保障改善民生为根本，推动公共服务均等化和社会治理科学化。完善公共服务和社会治理体系，办好10件48项惠民实事（见附件二），提高人民群众幸福感和满意度。

　　拓宽富民增收渠道。以创业带动就业、促进增收，强化创业载体建设和政策支持，争创省创业型城市。加强职业培训，落实小微企业吸纳当地劳动力就业扶持政策，促进高校毕业生、城镇困难人员、退役军人、“4050”人员就业以及困难行业下岗职工再就业，新增就业9万人。健全工资协商和正常增长机制，增加居民资产性、财产性以及转移性收入。落实支持经济薄弱村发展措施，培育土地股份制等富民合作经济组织，拓展农民增收空间。

　　健全社会保障制度。以更加公平可持续为目标，提高社会保障统筹层次和水平。完善城乡居民基本养老保险制度，有序将被征地农民纳入城镇职工养老保障体系，做好城乡居民、城镇职工大病保险工作，推进医疗、生育保险市级统筹。健全住房保障和供应体系，推动公共租赁房与廉租房并轨运行，扩大住房保障覆盖面；集中力量加快动迁安置房建设，缩短安置周期，保证安置质量。加强城乡居民最低生活保障规范管理，健全特困对象专项救助、自然灾害民生保险等制度，引导慈善组织、志愿者、企业等力量参与社会救助。

　　发展社会事业。推进省市共建教育现代化市级示范区，提高学前教育普惠水平，促进义务教育优质均衡、高中教育优质特色发展，深化职业教育产教融合、校企合作，支持高等教育开放合作、创新发展，加强特殊教育、继续教育，实施“智慧教育”优质资源共享工程。完善区域卫生规划，优化市区医疗资源配置，提升县级医院和基层医疗机构服务水平，推进“智慧卫生”协同创新，拓展与上海等地名院名医合作。健全以居家养老为基础、社区服务为依托、机构养老为支撑的养老服务体系，每千名老人拥有养老床位达到28.5张。促进人口长期均衡发展，启动实施“单独两孩”政策。推动群众体育和竞技体育协调发展。做好全国第三次经济普查工作。积极发展妇女、儿童、青少年、残疾人、关心下一代、红十字等事业。

　　促进文化繁荣。加快江海特色文化强市建设，提升区域文化软实力。发挥先进文化引领作用。围绕“三个倡导”，以家庭、社区、村镇等文化为抓手，广泛开展群众性文化活动，培育和践行社会主义核心价值观。弘扬新时期南通精神，光大精神文明“南通现象”。打造诚信南通。完善文明城市建设长效机制。提高公共文化服务水平。扩大公共文化资源有效覆盖面，推进乡镇（街道）综合性文化服务中心建设，新建一批村（社区）文化活动设施。实施市文化艺术中心项目。支持文化艺术精品创作。增强文化产业发展活力。培育传统特色文化产业，做强文化创意、家纺设计、数字出版印刷等重点园区，文化产业增加值占GDP比重超过4%。推进一批文化产业重点项目，超亿元文化企业达到18家。

　　加强社会治理。围绕共建、共享、共治，推进系统治理、依法治理、综合治理、源头治理，有效预防和化解社会矛盾。强化基层组织、基础工作和基本队伍，健全社区综合服务管理平台。积极发展基层民主，提升群众自我管理和服务能力。推进“政社互动”试点，支持行业协会、商会等社会组织承接政府转移职能。围绕争创平安中国示范区，完善立体化、现代化社会治安防控体系，建立最严密、覆盖全程的农产品质量和食品药品安全监管制度，强化安全生产隐患排查治理和预防控制，健全防灾减灾应急管理机制，加大依法管理网络力度。着眼军事需求，深化军民融合式发展。加强国防动员、后备力量建设和国家安全、双拥、民族、宗教、档案、保密等工作。

　　（十）以提升效能为关键，切实转变职能改进作风。围绕创造良好发展环境、提供优质公共服务、维护社会公平正义，建设法治政府、阳光政府、服务政府，提升政府治理能力和水平。

　　加强政务服务。严格执行首问负责、AB角、限时办结等制度，下决心简政放权，提升“三中心一平台”服务效能。完善政务服务体系，加强电子政务建设，促进四级便民服务网络功能拓展，提高行政运行和服务效率。强化科学发展、创新驱动导向，加大政策对接和创新力度，完善重点经济工作综合考核和政府系统“创新奖”评选办法。加强效能监察、执法监察和廉政监察，坚决制止行政不作为、慢作为、乱作为。

　　规范行政行为。加快建设法治南通，深入开展“六五”普法，促进依法行政、依法办事。加强规范性文件和重大决策合法性审查，争取成为有地方立法权的较大的市。深化行政权力网上公开透明运行，健全政务公开和新闻发布制度。推进行政执法体制改革。自觉接受人大及其常委会的法律监督、工作监督和政协的民主监督，重大决策前及时向人大报告、与政协协商，认真办好人大代表议案、建议和政协建议案、提案。强化审计监督，重视公众监督和舆论监督，运用和规范互联网监督。

　　改进工作作风。认真开展党的群众路线教育实践活动，深入落实中央八项规定和省、市委十项规定，坚决反对“四风”，健全“三解三促”等联系服务群众制度。严格落实党风廉政建设责任制，认真执行厉行节约反对浪费和公务接待管理的各项规定，推进财政预决算和“三公”经费公开试点。构建决策科学、执行坚决、监督有力的权力运行体系，加强公务员教育管理，依法严惩违法违纪行为，进一步树立“为民、务实、清廉”的良好形象。

　　各位代表，做好今年的工作任务艰巨、责任重大。让我们紧密团结在以习近平同志为核心的党中央周围，在省委、省政府和市委的坚强领导下，锐意进取、开拓创新，齐心协力、真抓实干，以“两个率先”的过硬成果谱写好中国梦的南通篇章！
